# Cluster randomised trial in the General Practice Research Database: 1. Electronic decision support to reduce antibiotic prescribing in primary care (eCRT study)

**DOI:** 10.1186/1745-6215-12-115

**Published:** 2011-05-10

**Authors:** Martin C Gulliford, Tjeerd van Staa, Lisa McDermott, Alex Dregan, Gerard McCann, Mark Ashworth, Judith Charlton, Andrew P Grieve, Paul Little, Michael V Moore, Lucy Yardley

**Affiliations:** 1 Primary Care and Public Health Sciences, King's College London, London, UK; 2 General Practice Research Database (GPRD) Division, Medicines and Healthcare products Regulatory Agency, London, UK; 3 Utrecht Institute for Pharmaceutical Sciences, Utrecht University, Utrecht, The Netherlands; 4 Division of Community Clinical Sciences, University of Southampton, Southampton, UK

## Abstract

**Background:**

The purpose of this research is to develop and evaluate methods for conducting cluster randomised trials in a primary care database that contains electronic patient records for large numbers of family practices. Cluster randomised trials are trials in which the units allocated represent groups of individuals, in this case family practices and their registered patients. Cluster randomised trials often suffer from the limitation that they include too few clusters, leading to problems of insufficient power and only imprecise estimation of the intraclass correlation coefficient, a key design parameter. This difficulty might be overcome by utilising databases that already hold electronic patient records for large numbers of practices. The protocol describes one application: a study of antibiotic prescribing for acute respiratory infection; a second protocol outlines an intervention in a less frequent chronic condition of public health importance, stroke.

**Methods/Design:**

The objective of the study is to implement a cluster randomised trial to test the effectiveness of an electronic record-based intervention at achieving a reduction in antibiotic prescribing at consultations for respiratory illness in patients aged 18 and 59 years old in intervention family practices as compared with controls. Family practices will be recruited from the practices that presently contribute data to the UK General Practice Research Database (GPRD). Following randomisation, electronic prompts will be installed remotely at intervention practices to promote adherence with evidence-based standards of medical practice. The intervention was developed through qualitative research at non-intervention practices. Data for outcome assessment will be obtained from anonymised electronic patient records that are routinely collected into GPRD. This protocol outlines the proposed study designs, data sources, sample size requirements, analysis methods and dissemination plans. Ethical issues are also discussed.

**Discussion:**

Results from this study will provide methodological evidence concerning the use of electronic patient records and databases for implementing cluster randomised trials in primary care. The study will also provide substantive findings in respect of electronic record-based interventions to reduce antibiotic prescribing in primary care.

**Trial Registration:**

Current Controlled Trials ISRCTN 47558792.

## Background

This protocol aims to develop an application of electronic patient records to the evaluation of health interventions, including their health impacts and effectiveness. We aim to provide 'proof of concept' of the feasibility and utility of implementing cluster randomised trials utilising electronic patient records in a large national primary care database. The specific objectives of the proposal are to develop, and confirm the feasibility of, a resource efficient method for implementing cluster randomised trials in public health and health services research by implementing a cluster randomised trial in a primary care database using routinely collected electronic patient records to evaluate patient outcomes. There will be two interventions that will build on our previous research; one application is in a common acute condition - antibiotic prescribing in respiratory illness; the other is in a less frequent chronic condition of public health importance - stroke. This protocol only concerns the intervention on antibiotic prescribing in respiratory illness. The research will provide guidance for the future conduct of cluster randomised trials using electronic patient records.

In cluster randomised trials, entire areas or health service organisational units are allocated to intervention or control groups, with outcomes evaluated for individuals within each cluster [1]. Cluster randomised trials (CRTs) are increasingly utilised in public health and health services research and are especially important in the evaluation health service and public health interventions [2]. CRT designs may be used to avoid problems of contamination. CRTs also facilitate pragmatic evaluation of the effectiveness of interventions delivered in routine practice settings. In addition, CRTs allow estimation of cluster level elements associated with the efficacy of the intervention. However, compared with studies in which an equivalent number of individual subjects are allocated, CRTs generally have reduced power because of the correlation of individual responses within clusters. The extent of such clustering is not easy to anticipate [1]. Another difficulty is that only small numbers of clusters may be allocated in CRTs because recruiting, intervening in, and collecting data from clusters may be costly [3].

In this proposal, we suggest that these difficulties may be overcome, to a certain extent, by implementing CRTs within the General Practice Research Database (GPRD), a primary care database that includes records from large numbers of practices. The database will provide a sampling frame for the study, it will also provide a mechanism for the electronic capture of data that describes case-mix at baseline and outcome measures pre- and post-intervention. This will be done by randomising, intervening in, and analysing data from family practices already contributing their electronic patient records to the database. The GPRD offers an excellent pre-existing sampling frame with large numbers of practices covering 5% of the UK population [4,5]. Studies conducted in the GPRD should have good external validity, covering a range of geographical and demographic settings and levels of risk. The GPRD also offers ongoing data collection for baseline and outcome measures for all registered patients, offering the potential to implement studies with greater power at lower cost. GPRD can now be linked individually and anonymously to other National Health Service (NHS) datasets. Currently, 304 GP practices in England are participating in this linkage (about 50% of GPRD). Data from the Hospital Episode Statistics (HES) and National Death Certificates (with date and primary and secondary cause of death) will be used for this study.

The substantive application for the research is in antibiotic prescribing. The problem of resistance to antimicrobial drugs is growing and appropriate prescribing of antimicrobial drugs is of great public health importance [6]. Respiratory tract infections (RTI) account for some 300-400 consultations annually per 1000 registered patients [7] and up to 60% of all antibiotic prescribing in family practice [6]. Giving antibiotics to patients with RTIs is often motivated by a concern to meet patient expectations [8] but antibiotics do not provide clinical benefit in a majority of RTIs [6,9-11]. These illnesses are usually brief and self-limiting, complications are unusual even without antibiotics, [6,12] and antibiotics may promote the spread of resistant organisms [13]. GPRD-based research has shown that antibiotic prescribing for respiratory infections in primary care declined between 1995 and 2000, [7,14] but since 2000 antibiotic prescribing for respiratory illness has stabilised [15]. There is a need to develop and implement interventions that endorse evidence-based antibiotic prescribing in family practice [6]. The age range 18 to 59 has been selected for study because the perceived, and actual, risk of serious infective complications is lower than at the extremes of age.

## Methods/Design

### Objective

To implement a cluster trial in GPRD in a common acute condition. This study aims to test the effectiveness of an electronic record-based intervention at achieving a reduction in antibiotic prescribing at consultations for respiratory illness in patients aged 18 and 59 years in intervention practices as compared with controls.

### Practices and allocation

Practices are being recruited through a letter of invitation from GPRD. A record will be maintained of the numbers of practices approached, recruited and analysed. Since data for all practices are collected into GPRD, it will be feasible to compare participating and non-participating practices through analysis of anonymised data in GPRD.

GPRD practices are allocated by minimisation, stratifying for region and list size. Allocation is at KCL to ensure allocation is separated from the process of practice recruitment. Patients will be all registered patients aged 18 to 59 years. There will be no other exclusion criteria, so as to optimise both internal and external validity [16].

### Intervention

Electronic prompts have been developed based on recommended clinical practice guidelines to be activated during consultations for RTI in the selected age range. The electronic prompts promote no antibiotic prescribing, or delayed antibiotic prescribing, instead of the immediate prescription of antibiotics for respiratory tract infections. The prompts specifically incorporate recommendations from the recent NICE guidelines on antibiotic prescribing in respiratory illness [6]. The research also builds on existing work that has identified barriers to reducing antibiotic prescribing [17-19] by designing prompts that briefly address common concerns (e.g. including messages providing evidence regarding likely consequences of delayed prescribing or not prescribing). During consultations with patients presenting with symptoms of respiratory tract infection, primary care professionals will see the prompts which remind them of recommended standards of care in RTI. The prompts will also provide them with supporting information and links to evidence that supports the recommendations, in a format suitable for printing out for patients when appropriate. The decision on whether to follow the treatment suggestions included in the prompt will be at the discretion of the GP. The GP will also be able to terminate display of the prompt at any time. There will be no intervention at control practices. The intervention phase will continue for 12 months at each practice.

The VISION software used by GPRD practices does not presently include any reminders on antibiotic prescribing, so the trial will compare outcomes associated with the new prompts as compared with care with no prompts.

### Intervention development

Intervention requires the development of prompts that encourage primary care professional adherence with recommended processes of care. The first year of the project has included a workstream to develop appropriate interventions consistent with the initial phases of the MRC framework for complex evaluations [20]. The format and content of the messages to be used in the interventions have been developed by a multi-disciplinary grouping comprising the research team and primary care professionals. Interventions are grounded in theoretical models of behaviour change [21,22] and informed by pre-existing evidence including systematic reviews [23,24] and national clinical guidelines as well as qualitative research. The development process was used to explore the extent to which electronic prompts can be used not only to remind GPs of recommended behaviour but also to convince them it will be beneficial and assist them with implementation. Tape-recorded interviews were carried out with a maximum variety sample of GPs (N = 30) from local non-GPRD practices with a variety of characteristics, to identify factors likely to influence successful implementation, and to pilot messages that have been identified as most likely to positively influence prescribing behaviour [25]. Thematic analysis was used to determine the range of likely responses to the proposed intervention and messages, which were then iteratively modified as necessary. The development and design of the prompts are reported in detail by McDermott et al. [25]

### Intervention implementation

Prompts will be downloaded automatically through the DXS Point-of-Care system. DXS (UK) Ltd collects data on usage of the information provided. In order to understand utilisation of the intervention, we will analyse fully anonymised practice-level data on usage of the electronic prompts that comprise the intervention.

Initial evaluation has shown that GPs may either begin their record of the consultation by initiating an antibiotic prescription, or by recording a medical code consistent with respiratory tract infection. The prompts are designed to be sufficiently flexible to be activated either by the start of an antibiotic prescription or by the specification of a medical code. 'Antibiotics' are defined as including all drugs in section 5.1 of the British National Formulary with the exception of anti-tuberculous and anti-leprotic drugs. Initially, prompt activation will be by means of medical codes.

### Outcomes and analysis

Outcome evaluation will be through analysis of routinely-collected GPRD data during a defined study period, while historical information will be used to assess the baseline characteristics of the study patients. Information routinely collected into GPRD for all registered patients includes medical history, use of medicines, hospitalisations and other resource use, smoking history, laboratory tests, letters from specialists or hospitals. GPRD also now links patients in GPRD to the English Hospital Episode Statistics, with detailed information on date, duration and reason for hospitalisation. Availability of data for all registered patients has potential to minimise biases from patient selection/recruitment.

Electronic patient records will be eligible for trial analyses if they describe patients who consult with acute respiratory tract infections, defined using pre-specified Read codes that identify conditions appropriate for study, and are aged 18 to 59 years at the date of the consultation. Pre-intervention GPRD analyses have already been reported [15]. Medical codes have been selected for RTIs including sub-groups of colds, rhinitis and upper respiratory infection; sore throat, pharyngitis and tonsillitis; influenza; laryngitis and tracheitis including croup and epiglottitis; acute sinusitis; otitis media and earache; acute bronchitis; and chest infection and pneumonia. The primary outcome will be the proportion of RTI consultations with antibiotics prescribed over 12 months; secondary outcomes will be age- and sex-specific rates of RTI consultation, age and sex-specific proportion of RTI consultations with antibiotics prescribed, and occurrence of RTI complications. We will use linked Hospital Episode Statistics (HES) data for English practices to evaluate hospitalisation with respiratory illness. Analyses will also be reported separately for each sub-group of RTI codes. In order to provide further insight into safety issues, family practices will be offered the opportunity to notify the study team prospectively of suspected adverse events, in fully anonymised format, during the course of the trial.

Outcomes will be measured through analysis of GPRD electronic prescribing records as described previously [14]. Antimicrobial drugs included will be those in British National Formulary chapter 5.1 excluding anti-tuberculous and anti-leprotic drugs. A maximum of one RTI consultation and antibiotic prescription on the same day will be analysed. Only first consultations within the same episode will be evaluated for the primary outcome, using a 10 day time window. Data for the intervention phase of the trial will be analysed from the intervention start date to 12 months later. Trial analyses will estimate the difference (95% confidence interval) in the proportion of RTI consultations with antibiotics prescribed between intervention and control groups after adjusting for age-group, sex, pre-intervention prescribing proportion. A cluster-level analysis will be implemented using the practice specific proportions as observations, with minimum variance weights to allow for varying numbers of consultations per practice [26].

### Sample size calculation

The sample size calculation is based on a comparison between the intervention condition, in which the new prompts are present, and usual care, in which no prompts are present. A cluster-level analysis of the practice specific proportions will be implemented. The sample size calculation therefore estimates the number of clusters (practices) required for the study. In a systematic review, quality improvement interventions were associated with reductions in antibiotic prescribing of between 7% and 12% (Ranji et al., 2006). The study therefore aims to detect differences of less than 7%. The selected age-range comprises about 55% of the registered population with about 1,000 registered patients per general practitioner and about 4,500 patients per practice. In data from Gulliford et al. [15], the age-standardised rate of RTI consultations in the 18 to 59 years range was 280 per 1,000 in women and 146 per 1,000 in men in 2006. This suggests there will be about 959 RTI consultations per year, per practice. We observed 1166 consultations in 5,647 person years, [15] consistent with 932 consultations per year per practice. Assuming 10% of consultations may be second visits, there may be 850 eligible consultations per practice. The proportion of consultations with antibiotics prescribed in 2006 was approximately 39% for all RTIs, having declined from 44% in 1997 (unpublished data). We assume that the coefficient of variation of this proportion between practices is 0.23 from Ashworth et al. [14], with alpha = 0.05 and power = 0.8. From Hayes and Bennett [27] equation 4, to detect a 5% difference in the proportion of consultations at which antibiotics are prescribed, 47 practices per group will be required. To detect a 6% difference 32 practices per group will be required. The GPRD includes more than 400 practices and a recent questionnaire of 386 GPRD practices found that 68% (262) were interested in participating in clinical trials. We plan to include 50 practices per group.

### Research ethics and governance

Hutton [28] suggests that consent in a cluster trial may be sought for three main reasons:

i) for the use of routinely held data;

ii) for the collection of additional data specifically for the study;

iii) for the offer or administration of an intervention.

The practices included in the present research already contribute anonymised electronic patient records to GPRD under an established governance framework. No additional data will be collected for this study at the individual patient level.

Implementing the interventions will require the randomisation of practices that are already participating in GPRD. Edwards et al. [29], and MRC [30] guidance on cluster randomised trials, distinguish two types of cluster randomised trials. Type A (or cluster-cluster) interventions are implemented for the whole cluster and consent is required at the cluster level from a guardian or gatekeeper. This is in contrast to Type B (or cluster-individual) interventions that are implemented at the individual participant level. These require active recruitment of individual participants within practices with a requirement for informed consent at the individual participant level. In Type B studies, selection of individual participants subsequent to the randomisation of the cluster may represent a potentially serious form of bias.

In the proposed research covered by this application, the trial intervention will be implemented at the cluster (practice) level through a modification to the practice information system. In trials of cluster-level interventions, consent should be obtained from the guardian of the cluster (usually the senior partner) on behalf of the cluster members (registered patients) [29,30]. The guardian's consent is regarded as ethically justified if the expected utility associated with the trial intervention is greater than the alternative [29]. The proposed interventions will encourage primary care professionals to adhere to nationally agreed, evidence-based, standards of care. Electronic prompts will provide practitioners with additional information during the course of consultations. However, all clinical treatment decisions remain at the discretion practitioners and their patients. Although electronic prompts provide information and advice concerning recommended standards of care, practitioners and patients remain free to jointly negotiate a chosen course of action during each consultation.

Analysis of outcomes will be through the analysis of routinely collected and anonymised GPRD data at the individual patient level.

We have convened a Trial Steering Committee (TSC) and a Data Monitoring and Ethics Committee (DMEC) with Independent Chairs and two/three independent members for each committee. The study was approved by London Surrey Borders Research Ethics Committee (09-H0806-81) and by the MHRA Independent Scientific Advisory Committee on Database Research (ISAC) (08-083).

## Discussion

### Anticipated outcomes

This study will provide evidence concerning the feasibility of implementing CRTs using the electronic records of patients with both acute conditions. The research will specifically provide evidence concerning the effectiveness of a strategy based on electronic prompts at enhancing effectiveness of care.

### Evaluation

As these studies will be among the first intervention studies implemented within GPRD, evaluation of the obstacles, barriers and facilitators to implementation of intervention research within GPRD will be an integral part of the study. We will evaluate views of staff at GPRD and practices using a questionnaire to ensure the anonymity of practices is maintained. Towards the end of the trial, an invitation email to complete an electronic evaluation questionnaire will be sent to all practices (both control and intervention groups). Fidelity of adherence with the intervention protocol, as well as feasibility and acceptability of interventions and trial participation, will be specifically addressed. Quantitative data will be supplemented by telephone interviews with a purposive sample to explore experiences of the intervention in more depth.

### Reporting, dissemination and implementation

We will prepare interim reports as well as an end-of project report. We expect to publish our findings in peer-review journals and make presentations at scientific meetings and conferences. The methodology developed through this project will have wide potential for application in future research and we expect that dissemination efforts will also be facilitated by data providers including GPRD and other databases. A key output from the research will therefore be methodological advice that will identify and analyse the component tasks of implementing a cluster trial through electronic patient records.

### Limitations

We recognise that the study will have limitations both with respect to the feasibility of the research and the validity of the results. One of the main purposes of the research is to evaluate the feasibility of conducting cluster trials in an electronic database. We will therefore document and report the processes of research that either facilitate or impede the conduct of these cluster trials. Cluster trials are susceptible to bias. However, the implementation of a cluster trial within an electronic database offers the opportunity to evaluate such biases because data may be analysed both for participating and non-participating practices. For example, the behaviour of professionals at control practices may be modified through their participation in the study even though they are not exposed to the intervention. This potential bias may be evaluated by comparing changes in practice at non-participating practices and participating control practices.

## Abbreviations

GPRD: General Practice Research Database; HES: hospital episode statistics; KCL: King's College London; MHRA: Medicines and Healthcare products Regulatory Agency; NHS: National Health Service; RTI: respiratory tract infection.

## Competing interests

The authors declare that they have no competing interests.

## Authors' contributions

MG developed the idea and drafted the protocol; TvS, PL, MA, LY, JC and AG contributed to the design and implementation of the study and contributed to the protocol; AD contributed to the implementation of the study and contributed to the allocation process and analysis plan; GM was responsible for developing recruitment procedures; LM and LY with PL and MM were responsible for developing the trial interventions. All authors read and approved the final version.
